# Estimated prevalence of mucopolysaccharidoses from population-based exomes and genomes

**DOI:** 10.1186/s13023-020-01608-0

**Published:** 2020-11-18

**Authors:** Pâmella Borges, Gabriela Pasqualim, Roberto Giugliani, Filippo Vairo, Ursula Matte

**Affiliations:** 1 Cell, Tissue and Gene Laboratory, Clinicas Hospital of Porto Alegre, Rio Grande do Sul, Brazil; 2 Experimental Research Centre, Bioinformatics Core, Clinicas Hospital of Porto Alegre, Rio Grande do Sul, Brazil; 3 Graduate Programme in Genetics and Molecular Biology, Federal University of Rio Grande Do Sul (UFRGS), Rio Grande do Sul, Brazil; 4 Genetics Laboratory, Biological Sciences Institute, Federal University of Rio Grande (FURG), Rio Grande do Sul, Brazil; 5 Department of Genetics, UFRGS, Porto Alegre, Brazil; 6 Medical Genetics Service, HCPA, Porto Alegre, Brazil; 7 Center for Individualized Medicine, Mayo Clinic, Rochester, MN USA; 8 Department of Clinical Genomics, Mayo Clinic, Rochester, MN USA

**Keywords:** Mucopolysaccharidoses (MPS), Estimated prevalence, Exome aggregation consortium (ExAC), Genome aggregation database (gnomAD), In silico analysis

## Abstract

**Background:**

In this study, the prevalence of different types of mucopolysaccharidoses (MPS) was estimated based on data from the exome aggregation consortium (ExAC) and the genome aggregation database (gnomAD). The population-based allele frequencies were used to identify potential disease-causing variants on each gene related to MPS I to IX (except MPS II).

**Methods:**

We evaluated the canonical transcripts and excluded homozygous, intronic, 3′, and 5′ UTR variants. Frameshift and in-frame insertions and deletions were evaluated using the SIFT Indel tool. Splice variants were evaluated using SpliceAI and Human Splice Finder 3.0 (HSF). Loss-of-function single nucleotide variants in coding regions were classified as potentially pathogenic, while synonymous variants outside the exon–intron boundaries were deemed non-pathogenic. Missense variants were evaluated by five in silico prediction tools, and only those predicted to be damaging by at least three different algorithms were considered disease-causing.

**Results:**

The combined frequencies of selected variants (ranged from 127 in *GNS* to 259 in *IDUA*) were used to calculate prevalence based on Hardy–Weinberg's equilibrium. The maximum estimated prevalence ranged from 0.46 per 100,000 for MPSIIID to 7.1 per 100,000 for MPS I. Overall, the estimated prevalence of all types of MPS was higher than what has been published in the literature. This difference may be due to misdiagnoses and/or underdiagnoses, especially of the attenuated forms of MPS. However, overestimation of the number of disease-causing variants by in silico predictors cannot be ruled out. Even so, the disease prevalences are similar to those reported in diagnosis-based prevalence studies.

**Conclusion:**

We report on an approach to estimate the prevalence of different types of MPS based on publicly available population-based genomic data, which may help health systems to be better prepared to deal with these conditions and provide support to initiatives on diagnosis and management of MPS.

## Introduction

The mucopolysaccharidoses (MPS) are a group of lysosomal diseases characterized by the deficiency of one of eleven enzymes involved in the breakdown of glycosaminoglycans (GAGs) which are constituents of the extracellular matrix. When there is a disturbance in their activities this leads to downstream consequences at the cellular level affecting multiple organs and systems. The MPS may be divided into different types according to the enzyme deficiency and the accumulated substrate (type I, II, IIIA, IIIB, IIIC, IIID, IVA, IVB, VI, VII, and IX). GAGs are constituents of the extracellular matrix, where impaired activities can lead to a spate of negative consequences both at the cellular and the physiological levels. Affected individuals usually have coarse facial features, cardiac and pulmonary problems, and, depending on the MPS type, bone dysplasia (dysostosis multiplex) and/or neurological impairment such as behavioural problems and developmental delay [1–3]. The severity of the diseases is variable, and individuals with MPS I, II, IVA, VI, and VII may benefit from market-approved enzyme replacement therapy, while there are novel therapies such as fusion proteins, gene therapy, and genome editing under investigation for several MPS [4].

Incidence and prevalence data are important to back up health system decisions and are necessary to calculate the cost–benefit of new therapies and treatment. Despite extensive molecular characterization having been done for the genes that encode the enzymes involved in these diseases with over 2,109 pathogenic variants reported in the Human Gene Disease Database (HGMD®) [5], there is still lack of specific epidemiology data on MPS. Newborn screening programs that include lysosomal diseases have arisen worldwide and may bring valuable information. However, such programs are still largely restricted to very few countries and most types of MPS are not included in the list of screened diseases [6, 7]. Population-based genomic data can help narrow the information gap, since now it is possible to rely on carrier frequency instead of the incidence of a disease among live births. However, care must be taken when using in silico predictors to classify genetic variants in order to have the most reliable data possible.

Herein, we used the frequency of potential disease-causing variants present in population-based genomic databases such as the Exome Aggregation Consortium (ExAC) [8]⁠ and the Genome Aggregation Database (gnomAD) [9], to estimate the prevalence of the different types of MPS after applying Hardy–Weinberg principles [10].

## Results

Table 1 shows the number of variants present in each database and after the merger, which ranged from 961 (*IDS*) to 2988 (*GALNS*). After subsequent filtering steps, these numbers were reduced, ranging from 31 (*IDS*) to 259 (*IDUA*) (Table 2). A detailed description of the excluded variants can be found in Additonal file 1: Table S1.

**Table 1:** Number of variants in each gene present in ExAC and gnomAD

MPS type	Gene	ExAC variants	gnomAD variants	Common	Retained variants**
MPS I	*IDUA*	1246	1439	680	2005
MPS II	*IDS*	300	920	259	961
MPS IIIA	*SGSH*	1188	1400	545	2043
MPS IIIB	*NAGLU*	640	805	397	1048
MPS IIIC	*HGSNAT*	598	1456	521	1533
MPS IIID	*GNS*	429	1116	404	1141
MPS IVA	*GALNS*	1390	2254	656	2988
MPS IVB	GLB1*	871	1322	564	1629
MPS VI	*ARSB*	407	1122	370	1159
MPS VII	*GUSB*	593	1067	519	1141
MPS IX	*HYAL1*	669	700	287	1082

*Variants may be associated with GM1 Gangliosidosis or with MPS IVB

**Retained variants represent unique variants after merging both databases

**Table 2:** Number of variants considered deleterious per category for each gene

	Frameshift**	In-frame insertion/deletion	Splice site**	Start loss	Stop gain**	Stop loss**	Missense**	Total**
*IDUA*	17–18	12	16–37	1	10–15	0–1	86–175	142–259
*IDS*	0	1	1–2	0	0	0	4–28	6–31
*SGSH*	8–14	7	5–7	0	4–14	0	73–194	97–236
*NAGLU*	11–20	2	6–10	1	8–16	0	87–176	115–225
*HGSNAT*	11	4	22–37	0	8–9	0	18–98	63–159
*GNS*	5	3	14–23	0	4	0–1	29–91	55–127
*GALNS*	11	7	14–26	1	10–11	0–1	57–187	100–244
GLB1*	12–13	3	18–34	1	11–13	0	67–161	112–225
*ARSB*	9–12	5	10–18	0	8–12	0	48–141	80–188
*GUSB*	11–13	6	17–27	2	13–14	0–2	62–160	111–224
*HYAL1*	12–13	8	1–3	1	8–9	0	57–107	87–141
All genes	107–130	58	124–224	7	84–117	0–5	588–1515	968—2059

*Variants may be associated with GM1 Gangliosidosis or to MPS IVB

**Numbers represent minimum and maximum frequencies. In the case of frameshift, stop gain or stop loss minimum frequency excludes variants in the last exon or located < 50 nucleotides upstream of the 3’ most splice-generated exon-exon junction. For splice site and missense variants, minimum frequency considers only variants deemed pathogenic by a consensus of all software packages

The number of variants excluded due to homozygosis ranged between 3 in *GNS* and *GUSB* to 113 in *IDS* (in homozygosis or hemizygosis); none of them were stop gain, stop loss, or start loss. The overall number of heterozygous canonical and non-canonical splice site variants considering all genes was 452, with 224 being considered deleterious by the in silico algorithms. One splice site variant could not be analysed by HSF nor SpliceAI (Additonal file 3: Table S3). In addition, 213 out of 218 frameshift and 188 in-frame insertions and deletions were considered deleterious. Variants that could not be analysed by SIFT Indel were excluded from further analysis. All variants considered deleterious by only one splice program as well as frameshift and nonsense variants in the last exon or located < 50 nucleotides upstream of the 3’ most splice-generated exon-exon junction were excluded from the calculations of minimum frequency. The number of variants considered deleterious in each category is shown in Table 2.

All 3,111 missense variants were analysed by five different in silico tools. A consensus on pathogenicity was reached for 588 variants, while 548 variants were classified as pathogenic by four tools and 382 variants by three.

The allele frequencies of each variant for a given gene were added together and considered as the minimum and maximum frequency of the deleterious recessive allele. This number was then used to calculate minimum and maximum prevalence of disease based on the Hardy–Weinberg equilibrium (Table 3). As the number of variants retained for *IDS* was very low (31 variants), the estimated frequency of MPS II must be viewed with caution. It is worth noticing that variants on *GLB1* can be associated either with MPS IVB or GM1 gangliosidosis.

**Table 3:** Estimated disease prevalence based on allele frequencies of potentially disease-causing variants for each gene

Gene	Disease-causing variants	CI in 100,000 (max)	CI in 100,000 (min)
*IDUA*	259	7.103–7.096	2.479–2.476
*IDS*	29	0.0108–0.0107	0.00014–0.00013
*SGSH*	236	2.365–2.363	0.4116–0.4112
*NAGLU*	225	1.532–1.530	0.366–0.365
*HGSNAT*	159	1.566–1.565	0.107–0.106
*GNS*	127	0.459–0.458	0.0549–0.0548
*GALNS*	224	2.363–2.361	0.25–0.25
GLB1*	225	1.677–1.676	0.456–0.455
*ARSB*	188	1.119–1.117	0.1761–0.1758
*GUSB*	224	1.144–1.141	0.2081–0.2078
*HYAL1*	141	0.4393–0.4388	0.1081–0.1079

*Variants may be associated to GM1 gangliosidosis or to MPS IVB. CI = Confidence interval

Only two of the 2,061 retained variants have frequencies over 0.001—p.(His356Pro) in *NAGLU* with 0.007993 and p.(Asp152Asn) in *GUSB* with 0.001153. After all five tier variant selections, maximum and minimum estimated disease prevalence was calculated based on global allele frequency (Table 3).

**Fig. 1:**
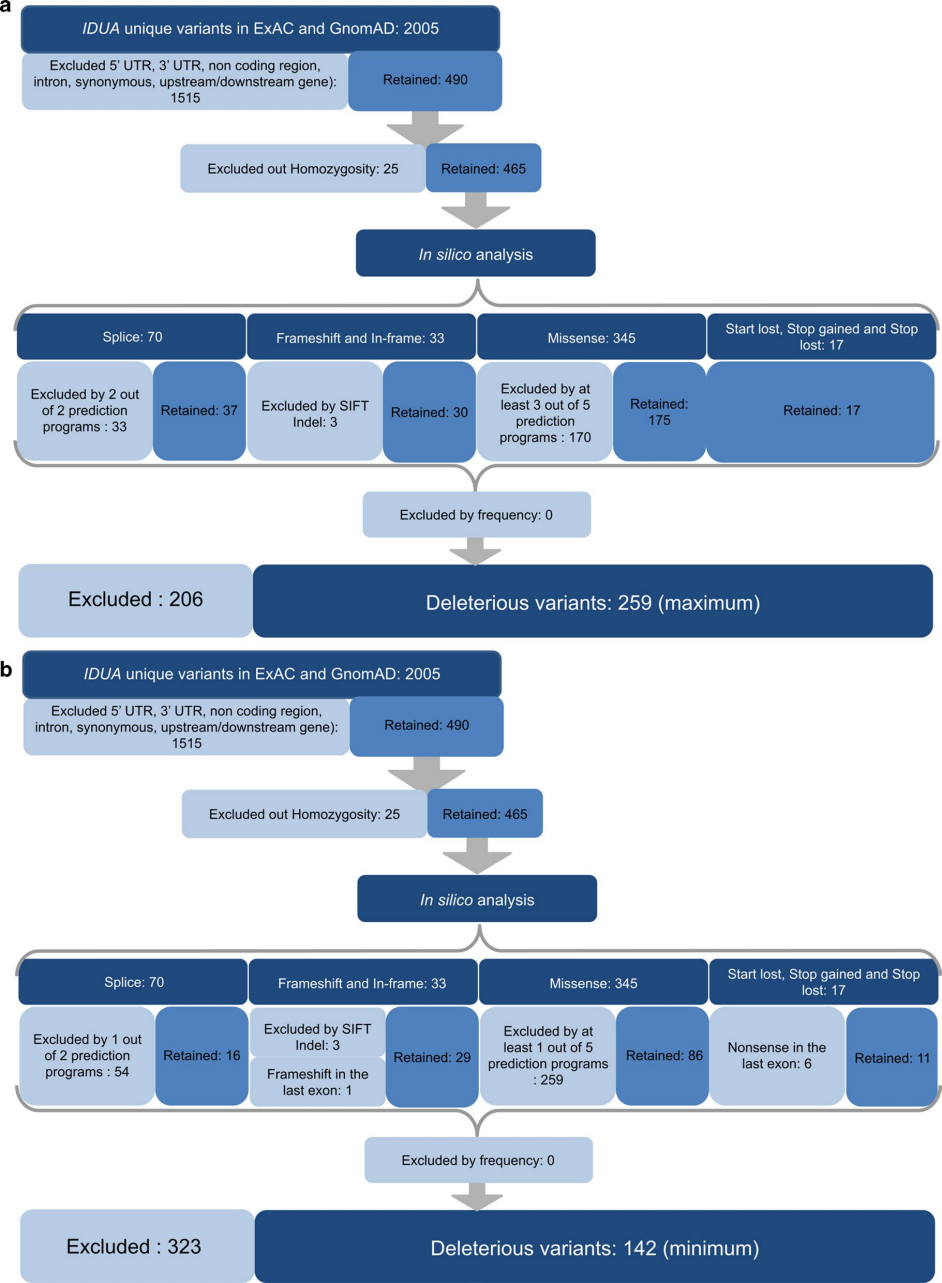
Schematic example showing all steps of maximum (**a**) and minimum (**b**) variant selection for the *IDUA* gene (MPS I)

**Fig. 2:**
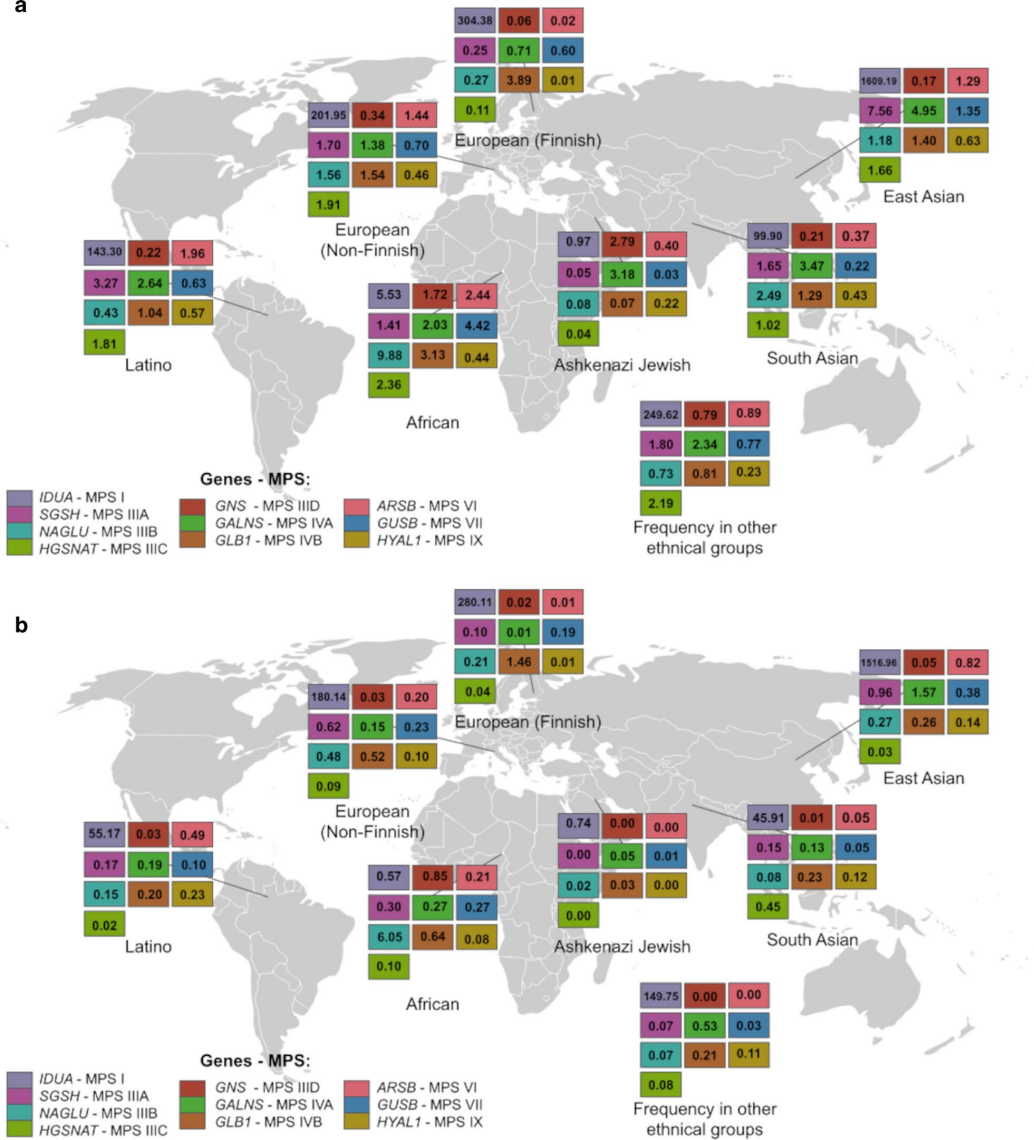
Estimated maximum (**a**) and minimum (**b**) prevalence of the MPS types per 100,000 individuals in different ethnic groups. Data for MPS II not included (see discussion)

In addition to estimated overall disease prevalence, the prevalence of MPS in specific populations was calculated for eight ethnic groups present in the databases (Figs. 1, 2 and Additonal file 4: Table S4).

## Discussion

In this study, we used public data from WES and WGS to estimate the prevalence of different types of MPS. As MPS symptoms usually show up in the first decade of life, it is unlikely that severely affected individuals would be part of such databases. However, the possibility of undiagnosed individuals with milder phenotypes being included in those cannot be ruled out. Importantly, individuals homozygous for rare variants present in any MPS gene (Additonal file 2: Table S2), which could represent individuals with attenuated forms of the disease were filtered out in the second-tier variant selection.

The estimated global frequency for all types of MPS except for type VI found in this study was either above or at the upper limit in comparison to frequencies of MPS in different countries based on the number of diagnosed cases in reference centres [20] (Table 4). Worthy of note is the fact that the maximum prevalence as reported by Khan et al., 2017 is for a limited number of countries, whereas our data was calculated collectively for the different ethnic backgrounds present in the databases. This means that we may have overestimated the prevalence of diseases in the general population. A recent study estimated the prevalence of MPS in Brazil based on 600 affected individuals with all types of MPS included in a national network database [21]. The researchers found discrepancy when comparing the estimated prevalence based on diagnosis (0.24/100,000) to the estimated prevalence based on genetic screening for the most common pathogenic variant in *IDUA* among healthy volunteers (0.95/100,000), for example. Furthermore, the estimated prevalence of MPS VI in Brazil was the second highest in the world, with prevalence similar to that found in the present study (1.02/100,000 compared with 1.12/100,000).

**Table 4:** Estimated prevalence in the present study compared to the incidence (in 100,000) as reported by Khan et al., 2017 for each MPS type

MPS type	Gene	This study (max.–min.)	Khan et al. 2017 (max.–min.)
MPS I	*IDUA*	7.10–2.48	3.62–0.11
MPS II	*IDS*	0.0108–0.00013	2.16–0.1
MPS IIIA	*SGSH*	2.36–0.41	1.62–0.08
MPS IIIB	*NAGLU*	1.53–0.37	0.72–0.02
MPS IIIC	*HGSNAT*	1.57–0.11	0.42–0.03
MPS IIID	*GNS*	0.46–0.05	0.10–0.09
MPS IVA	*GALNS*	2.36–0.25	1.30–0.15
MPS IVB	*GLB1*	1.68–0.46*	0.14–0.01
MPS VI	*ARSB*	1.12–0.18	7.85–0.02
MPS VII	*GUSB*	1.14–0.21	0.29–0.02
MPS IX	*HYAL1*	0.44–0.11	NA

*Combined frequency of GM1 Gangliosidosis and MPS IVB

Several measures were taken to reduce the chance of prevalence overestimation. For example, variants were filtered in sequential steps, in order to obtain the most specific data possible. Also, both homozygotes and variants with frequency higher than 0.001 were excluded. Additional filtering based on functional predictions was also performed in order to include only variants more likely to affect protein function. After that, all variants remaining for analysis had allele frequencies below 0.001 and most of them have not been previously reported as disease-causing. This was expected since variants classified as of uncertain significance (VUS) based on the standards and guidelines of the American College of Medical Genetics/Association of Molecular Pathology (ACMG/AMP) [10] are known to account for a substantial part of disease-causing variants for MPS and have a significant impact on incidence estimates. For example, Clark et al. [22] showed that 25% of VUS analysed in MPS IIIB were potentially disease-causing and cause reduced enzyme activity.

It is worthy of note that sequential filtering steps and use of consensus scores do not guarantee that only pathogenic variants are selected or that only non-pathogenic variants are discarded. However, the estimation error is not directly measurable. Furthermore, the high frequency filter is necessary to exclude variants with frequencies incompatible with MPS disease. Although this may lead the possibility of underascertainment, frequencies like 0.007993 and 0.001153 for variant c.1067A > C; p.(His356Pro) in *NAGLU* and the c.454G > A; p.(Asp152Asn) in *GUSB* are not found in clinical practice. These were the only two variants excluded because of high frequency. We considered using curated variants reported either on ClinVar or Human Genome Mutation Database (HGMD), however, this would significantly reduce the number of retained variants (for instance, from 259 to 47 for *IDUA*, data not shown). Different in silico tools were used to estimate the likelihood of a variant being disease-causing. However, as no data on the sensitivity and specificity of such softwares are available for MPS genes, it is impossible to estimate the number of false-positive results. For instance, several well characterized pathogenic variants reported in HGMD had low deleteriousness scores as evaluated by the Combined Annotation-Dependent Depletion (CADD) [23] that has an overall higher performance than other predictors (data not shown).

The existence of compound heterozygotes cannot be ruled out. In fact, most individuals with MPS who are not a result of from consanguineous marriage are indeed compound heterozygotes. However, due to the structure of both databases used in this study, it is impossible to set up conditions where the occurrence of variants in *cis* cannot be ruled out, which would contribute to the overestimation of disease prevalence.

Despite these limitations, a similar approach has been used by Appadurai et al., 2015 to estimate the prevalence of cerebrotendinous xanthomatosis (CTX). As in the present study, the authors suggested an apparent underdiagnosis of CTX based on the allele frequency of potentially disease-causing variants present in ExAC. Interestingly, the discrepancy between genomic data and the diagnosis-based incidence is more pronounced for the rarest MPS diseases, such as MPS IIIC, IIID, IVB, VII, and IX. For some forms of MPS I, II, VI, and IX, it is possible that variants leading to deficient enzyme activity are not clinically recognized due to attenuated phenotypes [24–26]. On the other hand, severe cases of MPS VII may lead to premature death before the diagnosis is reached or even sought [27].

Notably, data emerging from large datasets of WES and WGS are disclosing novel phenotypes for well-known diseases, especially intermediate phenotypes [28–30]. This may also be the case for MPS and could help explain the higher prevalence predicted by our work, with patients not being recognized clinically due to an unusual presentation.

In the case of MPS IVB, there is an additional complexity since the same gene is involved in another lysosomal disorder with different accumulated substrate and clinical features, called GM1 gangliosidosis [31]. In this study, variants of *GLB1* were considered disease-causing regardless of the associated phenotype. Therefore, the overall frequency of alleles was used to estimate the prevalence of MPS IVB, whereas in fact only about 13.3% of curated disease-causing variants in this gene are associated with MPS IVB, the rest leading to the three types of GM1 gangliosidosis [32].

After the filtering steps, *IDS* had a limited number of retained disease-causing variants (29 variants), and therefore the estimated prevalence for MPS II was lower than what has been previously reported [20]. The higher prevalence observed in studies based on reference centres and diagnostic laboratories may be related to the proportion of patients having de novo variants. Pollard et al. [33] show that this happens in 22.5% of MPS II cases. In addition, recombination events between *IDS* and its pseudogene *IDS2* are a common cause of the disease, with structural variants such as gross rearrangements and complete or partial deletions seen in between 10 and 28% of affected individuals [34–40]. Those types of variants could not be taken into account in our estimates because of the structure of the populational databases used. As a result, the estimated prevalence of MPS II is not as reliable as it is for the other types of MPS. It is worth mentioning that the other study that uses a similar method for two X-linked diseases (Menkes disease and *ATP7A*-related disorders) [41] also found a very low number of variants, which could suggest that this strategy is not the best approach for X-linked disorders.

## Conclusions

In summary, we report on an approach to estimate the prevalence of the different types of MPS based on publicly available population-based genomic data that may help to better tailor screening and diagnostic programs for these diseases, to prepare the health systems to deal with a more precise estimated number of patients, and may serve as a starting point for other rare-disease initiatives.

## Methods

### Database

*Genetic variants (GRCh37/hg19) from ExAC V0.3.1 and gnomAD v2.0.2 *[8, 9]*⁠ were used to estimate the prevalence of different types of MPS. These public data aggregated information from 125,748 WES and 15,708 WGS collected from unrelated individuals and 1,756 parent–offspring trios with no known rare disease. The genetic data were collected from case–control studies of adult-onset common diseases, spanning six global and eight sub-continental ancestries, determined by ancestry-informative markers* [9]*. Although related individuals can have an influence upon the frequency of variants, the size of the database which has a total of 141,456 individuals makes the influence of 1,756 trios irrelevant.*

The data was retrieved separately for each gene, and then merged to create one single unified database. When variants were common to both databases, the allele frequencies from gnomAD were used for further analysis, as it includes ExAC data.

### First-tier variant selection

Variants of the gene located in 5′ and 3′ UTR, upstream and downstream, as well as intronic and non-coding transcript exons, were excluded assuming that no disease-causing variant has been described in such positions for any MPS. In addition, synonymous variants outside the exon–intron boundaries were also excluded, as well as variants in non-canonical transcripts.

### Second-tier variant selection

In second-tier analysis, missense, nonsense, stop gain and stop-loss, frameshift, and splice site variants present in homozygosis (and hemizygosis for *IDS*) were excluded based on the assumption that neither ExAC and gnomAD include MPS-affected individuals as they exclude samples from patients with severe pediatric diseases and their relatives [8]. Therefore, any homozygous variant should not be pathogenic. Heterozygous loss-of-function variants such as stop gain, stop loss, and start loss were considered as potentially disease-causing, considering the impact on protein function and strong evidence of pathogenicity as per the ACMG/AMP guidelines [10].

### Third-tier variant selection

Heterozygous alterations in canonical or non-canonical splice site were analysed using Human Splice Finder [11] and SpliceAI [12]. In-frame insertions, deletions and frameshift variants outside the last exon were analysed using SIFT Indel [13]. Variants were classified based on the default algorithms parameters for deleteriousness.

### Fourth-tier variant selection

The analysis of missense variants was made using five in silico algorithms: MutPred [14], PolyPhen2 [15], PROVEAN [16], SIFT [17], and REVEL [18]. Since Polyphen2 provides more than two categories, results were transformed into binary data considering "possibly pathogenic" and “probably pathogenic” as deleterious. For REVEL, an ensemble algorithm, a rank score over 0.75 was considered deleterious. To calculate the maximum prevalence of the disease, a variant was considered deleterious when at least three software packages agreed on pathogenicity. For the minimum prevalence, we included missense variants for which all in silico tools agreed on pathogenicity.

### Fifth-tier variant selection

The remaining variants were analysed to make sure that only rare alleles were retained. Therefore any variant with a frequency greater than 0.001 was excluded, as no variants associated with low enzymatic activity (≤ 15% wild type) were found with higher allele frequencies [19].

### Calculation of disease prevalence using Hardy–Weinberg principles

The frequency of a given variant retained as being disease-causing was calculated by dividing the number of chromosomes bearing the genetic change by the total number of chromosomes subjected to analysis in this position. Then the sum of all variant frequencies for each gene was used as the frequency of the recessive allele (q). The prevalence was then calculated as q^2^, from the Hardy–Weinberg formula p^2^ + 2pq + q^2^. The incidence for each specific population was calculated using the population-specific frequencies.

### Calculation of confidence Interval

A script in R was used to estimate the confidence interval. The variances in the frequency of variants and in the prevalence estimate were calculated equally as exhibit eqautions 5 and 13 from Clark et al. [22]. The confidence intervals were adapted to consider the sum of allele frequencies instead of probability, as suggested by Clark et al. [22].

## Supplementary information

**Additonal file 1.**The number of variants excluded at each category for each MPS gene at the calculated maximums frequency. Bold numbers identify retained variants.

**Additional file 2.** The total number of variants excluded for homozygosis for each MPS gene and the number of homozygosis variants with frequency less than 0.001.

**Additional file 3.** The number of variants excluded from the analysis for each MPS gene.

**Additional file 4.** The number of variants excluded from the analysis for each MPS gene.

## Data Availability

The authors confirm that the data supporting the findings of this study are available within the article [and/or] its supplementary materials.

## Abbreviations

MPS: Mucopolysaccharidoses
GAGs: Glycosaminoglycans
HGMD: Human gene disease database
ExAC: Exome aggregation consortium
gnomAD: Genome aggregation database
VUS: Variants classified as of uncertain significance
CADD: Combined Annotation-Dependent Depletion
CTX: Cerebrotendinous xanthomatosis
WES: Whole exome sequencing
WGS: Whole genome sequencing
