# Supplementary material for: Estimated prevalence of mucopolysaccharidoses from population-based exomes and genomes
Source: Orphanet J Rare Dis. 2020 Nov 18;15:324. doi: 10.1186/s13023-020-01608-0 (PMC7672855; doi:10.1186/s13023-020-01608-0)
Supplement: Supplementary file 1 — Additonal file 1.The number of variants excluded at each category for each MPS gene at the calculated maximums frequency. Bold numbers identify retained variants. [file 13023_2020_1608_MOESM1_ESM.docx]

Sup Table 1. The number of variants excluded at each category for each MPS gene at the calculated maximums frequency. Bold numbers identify retained variants.

|  | *IDUA* | *IDS* | *SGSH* | *NAGLU* | *HGSNAT* | *GNS* | *GALNS* | *GLB1* | *ARSB* | *GUSB* | *HYAL1* | *All genes* |
| --- | --- | --- | --- | --- | --- | --- | --- | --- | --- | --- | --- | --- |
| Merged variants | 2005 | 961 | 2043 | 1048 | 1533 | 1141 | 2988 | 1629 | 1159 | 1141 | 1082 | 16730 |
| *Excluded in the first tier* |  |  |  |  |  |  |  |  |  |  |  |  |
| 5’UTR | 71 | 65 | 29 | 60 | 0 | 35 | 71 | 42 | 81 | 19 | 124 | 597 |
| 3’ UTR | 23 | 299 | 177 | 30 | 298 | 237 | 258 | 29 | 312 | 67 | 41 | 1771 |
| Upstream gene | 12 | 25 | 224 | 11 | 13 | 9 | 374 | 13 | 6 | 3 | 14 | 704 |
| Downstream gene | 6 | 1 | 286 | 192 | 7 | 4 | 292 | 8 | 7 | 8 | 367 | 1178 |
| Intronic | 834 | 197 | 244 | 140 | 375 | 321 | 521 | 770 | 215 | 246 | 94 | 3957 |
| Non coding transcript exon | 204 | 10 | 453 | 0 | 227 | 81 | 700 | 115 | 61 | 191 | 0 | 2042 |
| Synonymous | 186 | 100 | 94 | 171 | 133 | 124 | 147 | 146 | 122 | 179 | 117 | 1519 |
| Non-canonical transcript | 179 | 66 | 132 | 48 | 86 | 31 | 201 | 55 | 14 | 5 | 12 | 829 |
| **Retained variants** | **490** | **198** | **404** | **396** | **394** | **299** | **424** | **451** | **341** | **423** | **313** | **4133** |
| *Excluded in second tier* |  |  |  |  |  |  |  |  |  |  |  |  |
| Homozygosity | 25 | 113 | 14 | 7 | 11 | 3 | 19 | 9 | 7 | 3 | 10 | 221 |
| **Retained variants** | **465** | **85** | **390** | **389** | **383** | **296** | **405** | **442** | **334** | **420** | **303** | **3912** |
| *Excluded in third tier* |  |  |  |  |  |  |  |  |  |  |  |  |
| HSF* and spliceAI | 33 | 8 | 14 | 13 | 31 | 18 | 33 | 27 | 18 | 28 | 5 | 228 |
| SIFT Indel | 3 | 1 | 1 | 3 | 3 | 1 | 4 | 6 | 2 | 2 | 4 | 30 |
| **Retained variants** | **429** | **76** | **375** | **373** | **349** | **277** | **368** | **409** | **314** | **390** | **294** | **3654** |
| *Excluded in the fourth tier* |  |  |  |  |  |  |  |  |  |  |  |  |
| Consensus for non-pathogenicity | 77 | 20 | 80 | 75 | 80 | 64 | 40 | 92 | 71 | 108 | 82 | 789 |
| 4 out of 5 | 57 | 16 | 27 | 42 | 67 | 49 | 44 | 54 | 27 | 24 | 44 | 451 |
| 3 out of 5 | 36 | 9 | 32 | 30 | 43 | 37 | 40 | 38 | 28 | 33 | 27 | 353 |
| **Retained variants** | **259** | **31** | **236** | **226** | **159** | **127** | **224** | **225** | **188** | **225** | **141** | **2061** |
| *Excluded in the fifth tier* |  |  |  |  |  |  |  |  |  |  |  |  |
| Allele Frequency | 0 | 0 | 0 | 1 | 0 | 0 | 0 | 0 | 0 | 1 | 0 | 2 |
| **Retained variants** | **259** | **31** | **236** | **225** | **159** | **127** | **224** | **225** | **188** | **224** | **141** | **2059** |

*HSF: Human Splice Finder 3.0
