# Supplementary material for: Estimated prevalence of mucopolysaccharidoses from population-based exomes and genomes
Source: Orphanet J Rare Dis. 2020 Nov 18;15:324. doi: 10.1186/s13023-020-01608-0 (PMC7672855; doi:10.1186/s13023-020-01608-0)
Supplement: Supplementary file 2 — Additional file 2. The total number of variants excluded for homozygosis for each MPS gene and the number of homozygosis variants with frequency less than 0.001. [file 13023_2020_1608_MOESM2_ESM.docx]

Sup Table 2. The total number of variants excluded out homozygosity for each MPS gene and the number of homozigosity variants with frequency less than 0.001.

| **Homozygosity** | *IDUA* | *IDS* | *SGSH* | *NAGLU* | *HGSNAT* | *GNS* | *GALNS* | *GLB1* | *ARSB* | *GUSB* | *HYAL1* | ***Total*** |
| --- | --- | --- | --- | --- | --- | --- | --- | --- | --- | --- | --- | --- |
| In-frame insertion/deletion | 0 | 0 | 0 | 0 | 0 | 0 | 0 | 0 | 1 | 1 | 0 | **2** |
| Splice site | 4 | 9 | 1 | 1 | 1 | 0 | 4 | 1 | 1 | 0 | 0 | **22** |
| Missense | 21 | 104 | 13 | 6 | 10 | 3 | 15 | 8 | 5 | 2 | 10 | **197** |
| **Total** | **25** | **113** | **14** | **7** | **11** | **3** | **19** | **9** | **7** | **3** | **10** | **221** |
| Frequency <0.001 | 12 | 106 | 5 | 4 | 5 | 2 | 7 | 2 | 1 | 2 | 4 | 150 |
