# Supplementary material for: Estimated prevalence of mucopolysaccharidoses from population-based exomes and genomes
Source: Orphanet J Rare Dis. 2020 Nov 18;15:324. doi: 10.1186/s13023-020-01608-0 (PMC7672855; doi:10.1186/s13023-020-01608-0)
Supplement: Supplementary file 3 — Additional file 3. The number of variants excluded from the analysis for each MPS gene. [file 13023_2020_1608_MOESM3_ESM.docx]

Sup Table 3. The number of variants excluded by unprocessed for each MPS gene.

| **Unprocessed** | *IDUA* | *IDS* | *SGSH* | *NAGLU* | *HGSNAT* | *GNS* | *GALNS* | *GLB1* | *ARSB* | *GUSB* | *HYAL1* | ***Total*** |
| --- | --- | --- | --- | --- | --- | --- | --- | --- | --- | --- | --- | --- |
| Frameshift | 0 | 0 | 0 | 0 | 0 | 0 | 2 | 2 | 0 | 0 | 0 | **4** |
| In-frame insertion/deletion | 0 | 0 | 0 | 0 | 1 | 0 | 0 | 0 | 0 | 0 | 0 | **1** |
| Splice site | 1 | 0 | 0 | 0 | 0 | 0 | 2 | 3 | 1 | 2 | 2 | **11** |
| **Total** | **1** | **0** | **0** | **0** | **1** | **0** | **4** | **5** | **1** | **2** | **2** | **16** |
